# Together and Apart: A Gender‐Informed Qualitative Analysis of Childhood Brain Tumour and the Parental Relationship

**DOI:** 10.1002/pon.70274

**Published:** 2025-08-27

**Authors:** Kate Young, Christina Malatzky, Natalie Bradford

**Affiliations:** ^1^ Cancer and Palliative Care Outcomes Centre, Centre for Healthcare Transformation School of Nursing Faculty of Health Queensland University of Technology Kelvin Grove Australia; ^2^ Children's Brain Cancer Centre at the Centre for Children's Health Research Children's Health Queensland Hospital and Health Service Queensland Government South Brisbane Australia; ^3^ Australian Centre for Health Services Innovation (AusHSI) and Centre for Healthcare Transformation, School of Public Health & Social Work Queensland University of Technology Brisbane Queensland Australia; ^4^ Centres for Justice and Decent Work & Industry, School of Public Health and Social Work Faculty of Health Queensland University of Technology Kelvin Grove Australia; ^5^ Viertel Cancer Research Centre, Cancer Council Queensland Brisbane Queensland Australia

**Keywords:** Australia, brain neoplasms, cancer, family, gender role, oncology, paediatrics, parents, qualitative research

## Abstract

**Objective:**

This study aims to investigate the gendered impact of childhood cancer on the parental relationship, specifically on the work of parenting together.

**Methods:**

Parents/carers of a child diagnosed with a brain tumour and receiving care within a statewide hospital and health service in Australia were invited to participate in semi‐structured interviews about their associated experiences. Data were collected from February 2021 to December 2022. Interview transcripts were analysed using reflexive thematic analysis.

**Results:**

Twenty‐four parents/carers representing diverse socioeconomic backgrounds and brain tumour experiences were interviewed (mothers: *n* = 22). All parents in a relationship (*n* = 19) discussed the impact of their child's tumour on their relationship with their co‐parent. Gendered patterns were identified in coping styles and family roles; some participants described these as a source of struggle. Mothers were more often reported as doing ‘emotion work’ for their family; fathers were more often reported engaging in ‘emotion‐avoidant’ coping strategies. Parents consistently spoke of the need to be ‘together’ in the experience of their child's cancer, though this was rarely elaborated upon.

**Conclusion:**

Future research and clinical guidance must apply a gendered lens to highlight the ways that mothers are made responsible for the emotional wellbeing of others and support more equitable divisions for this responsibility, including ensuring fathers receive supportive care outside of the parental relationship amid childhood cancer.

## 1 Background

From when symptoms first appear, childhood cancer is a challenging experience for families [1, 2]. Effects on the parental relationship have received increased scholarly attention in the last 2 decades, but few studies have done so through a critical lens that explicitly considers the social construction of gender [1, 3]. An investigation of parents' experiences of having a child diagnosed with a brain tumour—the deadliest solid tumour for children worldwide [4]—provides an opportunity to develop understanding of this phenomena. Childhood brain tumour has unique challenges for treatment and long‐term care being located in an essential part of the human body at a pivotal point in cognitive, physical and social development [5]. The ∼75% of children who survive are at risk of ‘late effects’ including neurocognitive deficits, metabolic syndrome and mental health disorders [6], that have social and economic ramifications for the diagnosed child and their family [1, 7, 8]. These ‘late effects’ necessitate long‐term caregiving needs that are shouldered disproportionately by mothers [1, 3, 8, 9], reflecting the enduring construction of mothers as principally responsible for childrearing, and the broader under‐valued informal caregiving they conduct globally [10, 11].

The parental relationship often plays a central role in how families cope with childhood cancer [12], and the long‐term psychosocial wellbeing of the diagnosed child [13] and siblings [14]. Neugebauer and Mastergeorge [15], in their adaptation of the Family Stress Model to paediatric cancer, suggest parents who can navigate their child's diagnosis and associated ramifications together in a functional way are more likely to foster their child's psychosocial wellbeing. The model articulates several factors that influence this process, including social support and healthcare delivery. In a glaring omission, gender is not considered despite it affecting every part of the model: women are more likely to experience economic hardship, greater caregiver burden, and poorer psychological health [16, 17]. Gender also remains un‐articulated in paediatric cancer standards of psychosocial care [2]. It is, however, increasingly recognised in cancer care and research more broadly, with a recent Lancet Commission outlining how the historic gender‐blind approach has harmed women [18]: “patriarchy dominates cancer care, research, and policy making. Those in positions of power decide what is prioritised, funded, and studied.”

Previous scholarly investigations into the gendered impacts of childhood cancer on the parental relationship have been largely quantitative and/or descriptive. These have predominantly focused on coping strategies, role changes, distress, and impact on relationship satisfaction and cohesion [3, 9, 19, 20]. Where relevant cancer diagnoses are reported, brain cancer is often represented by varying numbers of participants, but few analyses focus explicitly on brain tumour and its unique challenges. We thus aimed to conduct a gender‐informed analysis of parents' reported experiences of the impact of childhood brain tumour on the parental relationship. In this paper, we use the term ‘parental relationship’ to denote the work of parenting together.

## 2 Methods

This study is part of a larger mixed‐methods project aimed at improving psychosocial and economic outcomes for families who have a child diagnosed with a brain tumour. These methods included a 2‐year longitudinal survey [21], interviews (see, also, Young et al. [22]), and a community art exhibition [23]. The current study draws exclusively on the interview data.

### 2.1 Theoretical Framework

This paper was conceived within a social constructionist theoretical framework through a feminist lens [24], centring on the notion that knowledge is created through humans' collective assumptions about the world as shared through language, and that there is no ‘true’ reality of the world to be discovered [25]. In applying this lens, we intend to make transparent how gender influences the experience of childhood cancer for parents and explore the implications for their psychosocial wellbeing.

### 2.2 Sample and Recruitment

The study hospital is a state‐wide public paediatric tertiary facility in the state of Queensland, Australia. All children (0–14 years) and most adolescents (15–18 years) in this state diagnosed with cancer—approximately 300 per year—receive centralised oncology care through this service; approximately 65 with a central nervous system (most often brain) tumour are referred annually. Any parent or carer (a person who is not the biological parent of the child but is the legal guardian and/or primary carer of the child) aged at least 18 years with a child receiving care within this service for diagnosis or progression of a malignant or non‐malignant brain tumour between 2019 and 2022 were eligible to participate. Due to a lack of translation services, all participants had to be English‐speaking.

Possible families were identified by our clinical research nurse at weekly Solid Tumour Service Multi‐Disciplinary Team meetings and in consultation with treating clinicians. When deemed appropriate, they approached a parent/carer to invite them to participate in the study (longitudinal survey and/or interview), following established recruitment principles [26]. If interested, parent/carer's written informed consent was collected and a time for them to be interviewed arranged.

### 2.3 Data Collection

A semi‐structured interview guide was developed, informed by a recent systematic review [1], the study aims, and feedback received from Brainchild (brainchild.org.au)—an Australian consumer advocacy group for childhood brain tumour who reviewed the guide (see Online Supplementary File 1). Consistent with constructivist approaches to qualitative interviewing, this guide was used as a scaffold to prompt conversation when stilted; participants were encouraged to lead the direction of inquiry as to what was central to their experience. One such prompt explicitly asked participants about the impact on the parental relationship: *How has this experience impacted on your relationship with your partner [or your child's mother/father]?* Interviews were audio‐recorded and could be conducted by telephone, online video call, or in person (in hospital or at the co‐located research facility). Due to policies implemented during the initial phases of the Covid‐19 pandemic that inhibited non‐clinical research staff from entering the hospital, Author A trained our clinical research nurse to conduct most (*n* = 21) interviews, with Authors A and B conducting the remainder. Participants could be interviewed alone and/or with their co‐parent/carer. To reduce participant burden, demographic information was collected from hospital records. At the conclusion of each interview, participants were invited to email the study team if they wanted to arrange a follow‐up interview. Data were collected from February 2021 to December 2022.

### 2.4 Data Analysis

To explore themes of importance in the context of the gendered social world within which we exist, we analysed the data using reflexive thematic analysis with an inductive approach to both semantic and latent coding [27, 28]. Interviews were transcribed verbatim. Author A had read all interview transcripts several times for a previous unrelated analysis [22]—she read them again with a focus on parental relationships prior to beginning this specific analysis, noting initial ideas for themes that she then discussed with Author C. Author A then entered all transcripts into NVivo [29] and coded broadly for ideas that captured a facet of participants' experiences relevant to the research question. Once all transcripts were coded, Authors A and C re‐assessed theme groupings and rearranged them, where necessary, to reflect the entire dataset. In writing up the findings of the analysis for this publication, themes continued to be refined and discussed with the author team.

### 2.5 Reflexive Statement

This body of work reflects in part our views and experiences of the world [30]. We have each experienced unearned privileges in the country now known as Australia as non‐Indigenous people with settler ancestry. We have also experienced economic and educational privileges to be in the position to produce this work. We all identify as women and are all mothers. Author A is a social and behavioural scientist who has worked in childhood cancer research for 5 years. Author B is a feminist sociologist and critical social science scholar; this is her first work in childhood cancer. Author C has a clinical nursing background specialising in paediatric oncology and palliative care but has not practised for several years; she has worked in childhood cancer research for over 2 decades.

### 2.6 Ethics

The study protocol was approved by the hospital's Human Research Ethics Committee HREC/19/QCHQ/53816. We report descriptive information as a collective and describe aspects of care broadly (e.g., ‘health professional’ rather than exact profession) to preserve the privacy of families; a relatively rare brain tumour diagnosis and care within this known hospital and health service puts families and clinicians at higher risk of identification.

## 3 Results

One‐hundred and thirty families were approached to participate in the larger mixed‐methods study; 32 families declined to participate. Twenty‐two participants completed one or more surveys and one or more interviews; two participants completed an interview only. In total, 24 parents/carers were interviewed; one being a mother and one being a father from the same family—interviewed together for the first interview and the mother alone for the second. Demographic information for participants is outlined in Table 1. Six (25%) mother participants elected to a second interview 5–13 months after their first. Most (88%) participants were the mother. There was roughly equal representation of low‐ and high‐grade brain tumours. At the time of interview, no participant's child had died. Five participants (21%) referred to the ethnicity or cultural background of themselves or someone in their immediate family within their interview; broadly these were: Indigenous Australian, East Asian, North African, South American, and South African. The interviews ran for an average of 51 min (range: 20–107). Most interviews were conducted by telephone (*n* = 13; 45%), followed by video call (*n* = 11; 38%) and in person (*n* = 5; 17%).

**TABLE 1:** Demographic information for participants.

Variable	Characteristics
Parent interviewed	
Mother	21
Legal guardian/carer (female)	1
Father	2
Age of child at diagnosis, median years (range)	5 (3 months–17 years)
Time since diagnosis at interview, median months (range)	20 (2–61)
Siblings	
Yes	22
No	1
Relationship status	
Partnered	19
Single	5
Brain tumour grade	
Low grade	12
High grade	11
Home location in relation to hospital	
< 50 km	14
> 50 km	9
Household income	
< AU$70 000	8
≥ AU$70 000	10
Prefer not to say	2
Missing	3

*Note:* Totals vary due to one participant being a mother and one participant being a father from the same family.

We use the terms ‘wife’ and ‘husband’ if this was the term the participant used; otherwise, we use the term ‘partner.’ All participant names are randomly selected pseudonyms. We use the term ‘parents’ to denote individual participants rather than parental dyads, unless otherwise explicitly stated. The impact of their child's diagnosis on the parental relationship was a significant matter for many participants; some spontaneously discussed this early in their interview, while others in response to being asked. Five parents—all single mothers—made no mention of the impact of their child's cancer on the parental relationship; the father was not discussed at all (*n* = 3) or described as doing minimal parenting (*n* = 2). Our analytic review process resulted in a final structure of four themes that conceptualise how parents navigated their child's brain tumour within the work of parenting together and the gendered patterns weaved within this; these are outlined in Table 2.

**TABLE 2:** Themes and descriptions from a reflexive thematic analysis examining gender and the impact of childhood brain tumour on the parental relationship.

Theme	Description
I went one way, he went the other: Conflicting and converging coping styles	Parents often had conflicting styles in how they coped with their child's diagnosis, treatment and beyond. There were gendered patterns observed in information seeking and in how they addressed the experience post‐treatment. Conflicting coping styles often caused tension between parents; similar styles, however, did not shield parents from all challenges in how they worked together.
Boys are a hard egg to crack: Mothers supporting fathers' emotional wellbeing	Mothers discussed taking on the role of supporting fathers' emotions—who in this study more often had traumatic experiences of a loved one having cancer—while fathers' reciprocal support for mother's emotional wellbeing was absent.
This is my realm, this is your realm: Navigating roles as parents	Parents spoke of various factors that shaped their caregiving roles throughout their child's brain tumour experience: access to paid leave, support networks, and affordable accommodation. More often parents ‘tag teamed’ their roles—this was shaped by the child's age (e.g., an infant that was breastfed). Mothers only spoke of assuming responsibility for follow‐up therapies.
I just want to mention I love you: On being together	Being together in the experience was deeply valued by parents, though what was meant by ‘together’ was rarely explored. Brief emotional ‘check ins’ provided connection in challenging times.

### 3.1 I Went One Way, He Went the Other: Conflicting and Converging Coping Styles

For some parents, the way in which they coped with their child's illness compared to how their partner coped was a central theme in their interview. Ruth, for example, summarised:

I think if someone had just explained it to me that he processes things different than I do…that would've made it a lot easier, because…we didn't know that. I'm expecting him to feel one way, he's expecting me to feel one way, and he's dealing with it completely different than I'm dealing with it.

These different coping styles caused significant friction in Ruth and her husband's relationship for several months after their child's diagnosis. Ruth expressed a need to focus on her unwell child, while her husband expected her to also check in with his needs. However, at difficult points when Ruth communicated that she was not okay, she felt her husband was dismissive of her feelings because he did not want to acknowledge the severity of their daughter's condition—an observation that several mothers made of their male partners, more so in the aftermath of treatment, discussed further below. Their relationship improved with the support of friends and mental healthcare, and they found acceptance in that they were “*never going to be lined up.”* Seven months later in her second interview, Ruth spoke about how “*because we've done those hard yards*” she and her husband had learned to communicate with each other when they were struggling, prompting one to take over more childcare so that the other parent can “*crash*.”

Lizzie's description of her and her husband's dissimilar coping styles—and broader experiences of their child's illness—reflected an intersection between their different genders and cultural backgrounds. She described not feeling that her husband was “*useful*” while their daughter was in treatment as “*he wasn't talking, he was just in prayer a lot*.” Lizzie perceived some hospital staff may have viewed their relationship as concerning due to her husband's demeaner and cultural practices. She understood why “*they might have been concerned,”* but felt this was “*pressure on top of what we were going through*” and articulated a need for staff to consider that “*when you're in the hospital with a sick kid, everything's not going to be the same as it is at home*.”

Information preferences were a common point of difference for parents with a gendered pattern. Mothers were typically described (by themselves and/or their partner) to find more information about their child's diagnosis and treatment from varied sources as useful, while fathers were described (by themselves and/or their partner) as preferring a small amount from one source, usually their treating doctor: *He saved up all his questions for when he sees* [doctor]…*it was rock solid intel … He found that comforting, whereas I was happy to take it* [information] *from everywhere* (Rose). Parents sometimes attributed this difference to the mother having a health or science background while the father did not.

To a lesser extent, some mothers reported that they and their partner had similar coping styles. Madeline, for example, described how “*we're both really practical people…we're not overly emotional people*.” Diane reported her and husband to be “*very similar…how we deal with crises is very much shut everything off and just control what you can*.” Having similar coping styles, however, did not necessarily reduce the impact on the parental relationship with other influential factors at play, discussed further below.

For those who had returned home after the completion of treatment, several spoke of having different needs in processing the experience. Mothers more often discussed a need to reflect and understand what had happened, and to contemplate the implications for their future and that of their families. Comparatively, fathers were more often described as wanting to move on and put the experience behind them, believing “[child] *is better now, everything's been sorted*” (Rose). Cicely, for example, wrote a book about their experience that “*turned into a wonderful project for me…my healing, my continued mental health*” and that this has helped her face the challenges with her child's unexpected ongoing treatment. She expressed that her husband was initially “*reluctant*” to read it as he didn't want to relive “*the really hard times*.”

### 3.2 Boys are a Hard Egg to Crack: Mothers Supporting Fathers' Emotional Wellbeing

Mothers more often reported actively supporting their own mental health and emotional wellbeing by seeking associated care for themselves early after their child's diagnosis or after treatment was completed. Some also spoke of supporting their male partner's mental health by encouraging them to seek informal support through friends and family, or formal support through a psychologist or counsellor:

I tried when I was seeing a counsellor…why don't you go? He's a very typical bloke that is just like…I'm good…but are you?…there's definitely things that happen that I'm like, maybe you should talk to someone about that…but boys are a hard egg to crack, aren't they?—Diane.

No participant spoke about a father encouraging a mother to access such support. In our sample, it was notable that fathers more often had experienced a close relative having cancer—parents attributed this formative experience as influencing their supportive needs for their child's cancer: *His mum had…cancer and she died when he was* [a young adult]…*when we were then given the diagnosis of* [child's] *cancer, he straightaway just went…we're going to lose our son*” (Olivia).

The two fathers we interviewed both recounted seeking mental health support. Taylor, for example, explained that while not being “*a person who speaks very often about my emotions*” he did reach a point where “*I was just thinking I was losing my stuff*” and he and his wife were “*finding it tough in our relationship*.” They sought help from a hospital staff member who he recalled saying, “*I wish I could get my husband to do this*.”

### 3.3 This is My Realm, This is Your Realm: Navigating Roles as Parents

Parents spoke about the challenge of co‐navigating their child's care while also meeting the needs of their other children and maintaining paid employment. Some mothers described the father as the one who had to do much of the “*leg work*” for diagnosis and treatment due to the logistics of having an infant: *We had a newborn baby and another child…so my husband took her* [diagnosed child] *on his own* (Bernadette), and Rose shared, “*It was tough. Breastfeeding and managing the kids at home and trying to juggle all the balls*.” One mother, Diane, shared that while her husband did try, their daughter “*didn't want him, which I completely get when you're three*” so “*I had to sleep in the hospital for like 3 months*.” Mostly, parents spoke about ‘tag teaming’ to take turns to be with their child in hospital, and to a lesser extent, caring for them when at home.

The decision‐making about which parent would do what was rarely explicitly described within parents' interviews. Various influential factors were referred to, with their impact changing at different points in the illness trajectory: access to paid leave, family support to assist in caring for siblings, and affordable accommodation for those who lived far from the hospital. Two roles more explicitly discussed were information seeking and recording, and the organisation of post‐treatment hospital appointments and therapies.

Mothers were more often described as the ones who sought out and managed information about their child's diagnosis. Walter, for example, described in their shared interview how his wife “*was keeping notes the whole time*” whereas “*I do not want to read into this because I'm going to get more upset and I put trust in the systems around me*.” His wife, Jane, described how, early after diagnosis while she was “*still in shock,*” she felt better “*able to compartmentalise*” and thus “*took on a lot of the admin,*” explaining that they had together “*talked it through…this is my realm, this is your realm*.” Jane described Walter as doing “*the hard yards*” with taking their child in for treatment every day for several weeks as that was “*his realm he's very good at*,” something that Walter attributed to the bond he had grown after taking on more of her care with the recent birth of a younger sibling prior to her diagnosis.

For those with a child who had completed treatment, many mothers spoke about the intense mental load of facilitating follow‐up hospital appointments and therapies. Olivia described managing hospital appointments as a “*juggling act*” for her and her husband, with one taking the day off paid work and the other finishing early to collect their other child from school. More commonly, it was only mothers who facilitated this aspect of care. Lizzie, for example, when asked if her husband assisted with post‐treatment appointments responded, “*No, he doesn't. I just do it all*,” despite her being the only parent in paid employment. Diane shared how she organised all government support and therapies for their child citing her familiarity with bureaucratic systems as skilling her in the process: *If I gave him* [husband] *the form he'd be like, ‘stuff that, I'm not writing the same sentence six times…it's too hard*.’ Despite being back at “*almost full‐time hours*” she shouldered this aspect of care, as her husband worked night shifts and was less available at relevant times:

*I'll have to jump out of work 3 days in a row and take her to those sessions…sometimes I just feel like there's so much to try to fit in because we're trying to set her* [daughter] *up as well as possible…when he's* [husband] *on days off, he tries really hard, but…it's still very traditional in that I do groceries and most of the cooking and organise all the house admin stuff*.

### 3.4 I Just Want to Mention I Love You: On Being Together

Parents frequently stated it was important they be ‘together’ with their partner in the experience of their child's illness, but there was little description of what this meant to parents or how they practiced it. Nonetheless, we report this theme due to the weight it held for participants.

Most expressed a similar perspective to Scarlett in that the “*tough times always make you more resilient as a couple*.” However, some stated “*there's very few couples still together*” (Cicely). After working through significant relationship challenges for much of their daughter's treatment, Ruth and her husband came to a decision that “*we need to be together on this and we need to understand each other are going through things, not just one of us*.” In her follow‐up interview, she described how they continue to “*work as a team*” through ongoing challenges. Some mothers, such as Bernadette, simply stated that they felt they and their partner “*did a really good job*” managing the experience together.

The end of treatment and returning home did not mean the end of challenges for families and the parental relationship. Diane, for example, spoke about how “*it's really busy*” juggling multiple therapies on top of paid work and other family needs, and that their “*relationship is not our top priority at this point in time*.” Even so, they still “*have those moments, like, I still love you…*[we still] *pinch each other on the bum and stuff like that*.” The benefits of these brief ‘check‐ins’ and displays of affection was echoed in Jane's follow‐up interview when they were facing an unknown time with their daughter before she would die from her cancer:

One of us will say to the other one, I just want to mention, ‘I love you…we're just in a really shit situation’…and it just keeps you going.

## 4 Discussion

Consistent with previous research in childhood cancer [3, 19], there were gendered patterns in reported coping strategies. Mothers more often described engagement with their emotions, while fathers were more often described attempting to contain the situation through, for example, minimal information from a consistent source and compartmentalising the experience to their past once treatment finished. Repressing emotions and disengagement from processing the experience is a consistent finding for fathers internationally [31, 32, 33]. The use of ‘emotion‐avoidant’ strategies by fathers may result in mothers shouldering this for them [19], as reported in our study. This reflects Duncombe and Marsden's [34] ‘gender division of emotion work’ that positions women as responsible for managing emotion within the family. In recent studies of serious childhood illness, however, fathers have challenged the social expectation to repress emotions [35, 36, 37], suggesting social shifts are occurring.

Our findings suggest fathers are doing more caring work during the treatment phase in childhood cancer compared to that reported in earlier studies [9, 19], reflecting Australian trends where men overall are doing more unpaid domestic work than they have in the past [38]. After treatment, however, there was little discussion of fathers contributing to the work involved in managing follow‐up appointments and therapies. Mothers are overwhelmingly the primary caregiver for their child after treatment, including into adulthood in this study and others [39, 40]. While mothers may value their caregiving work, they maintain their right to their own wellbeing, and their social and economic participation [41, 42]. Mothers of a child diagnosed with cancer, for example, are at increased risk of suicide attempt up to 7 years post diagnosis while fathers are not [43]. Disproportionate division of caregiving is influenced by gender attitudes and women's position in the labour force; however, comparative modelling across 13 European countries suggests the most influential factor is the availability of public care services [44]. These are notably lacking for paediatric survivorship care, including into adulthood, globally [45].

Parents often spoke about the importance of being ‘together’ in the experience of their child's cancer. However, we did not elicit in‐depth descriptions of what ‘together’ meant and whether this differed by gender. Parents' accounts of using ‘check‐ins’ with each other resembles Gottman and colleagues' concept of ‘bids’—requests from one partner to another for connection that can be verbal or non‐verbal and vary in size and theme [46]. Parents use of these bids served to maintain connection during an overwhelming time.

### 4.1 Strengths and Limitations

Our sample was varied in terms of socioeconomic background and experience of childhood brain tumour; however, we had few fathers volunteer—a known phenomenon in childhood cancer research [37] and health research more broadly [47]. It is possible that the two fathers who did participate reflect those who are more involved in their child's treatment and care [3]. It is a limitation of this study that data on sexual orientation and identity was not explicitly collected; we had no parents who self‐identified as LGBTQI+. Research is needed into the experiences of parents and carers who identify as LGBTQI+, and for single‐parents, to gain a comprehensive evidence‐base. Our analysis and interpretation of data was strengthened by our research team having a diverse range of clinical and academic backgrounds. However, due to COVID‐19 pandemic restrictions, most interviews were conducted by our clinical research nurse rather than our experienced qualitative researcher. This potentially limited further elicitation of experiences and perspectives on the current paper's topic.

### 4.2 Clinical Implications

The Standards for the Psychosocial Care of Children with Cancer and their Families outline the need for comprehensive care for the whole family from diagnosis [2]. However, little consideration is given to gender, despite it being essential for the provision of safe care that reflects the social world families experience childhood cancer within [16, 17]. Our findings highlight the importance of clinicians being mindful of potential gendered influences on parents' experiences and supportive care needs, while not perpetuating and reinforcing these. Reported support interventions for parents of children with cancer are largely focused on improving individual psychological wellbeing with little acknowledgement of the parental relationship or gender; and they are overwhelmingly tested on mothers [48]. Based on our findings, we suggest such interventions incorporate (1) support to identify coping styles and to manage different or conflicting styles; (2) a ‘division of labour’ checklist specific to childhood cancer to facilitate conversations around equitable caregiving between parents/carers, including after treatment [49], and (3) suggestions for practical, low‐resource ways to foster connection between parents/carers amid trauma and/or physical separation.

## Author Contributions

**Kate Young:** conceptualisation, methodology, validation, formal analysis, investigation, data curation, writing – original draft, writing – review and editing, project administration. **Christina Malatzky:** methodology, formal analysis, writing – review and editing, supervision. **Natalie Bradford:** methodology, validation, formal analysis, investigation, resources, writing – review and editing, supervision, project administration, funding acquisition.

## Conflicts of Interest

The authors declare no conflicts of interest.

## Supporting information


Supporting Information S1
