# Supplementary material for: Together and Apart: A Gender‐Informed Qualitative Analysis of Childhood Brain Tumour and the Parental Relationship
Source: Psychooncology. 2025 Aug 27;34(9):e70274. doi: 10.1002/pon.70274 (PMC12383243; doi:10.1002/pon.70274)
Supplement: Supplementary file 1 — Supporting Information S1 [file PON-34-e70274-s001.docx]

**INTERVIEW QUESTION BANK - PRIMARY CARER**

| Participant | Interview questions |
| --- | --- |
| Diagnosis | |
| Primary care giver | Diagnosis   - Please tell me how you found out that [child’s name] had a brain tumour. *Prompts:* - What are some feelings that you had at the time? - What were some of the helpful things that the health care providers you were seeing did at this time? - What are some things that the health providers you were seeing could have done better at this time? - Can you recall any coping strategies that you used at the time for you and your family? *Prompt*: For example, reaching out to family and friends, searching for information.   Expectations for care   - Do you have any expectations for the healthcare and support that your child and family will receive over the next 12 months? If yes, what are they? *Prompt*: For example, which medical and health professionals do you think you will see in hospital? What kind of care and support do you hope to receive when at home? - Is there anything that you think might happen to prevent your child and family from receiving the care they most need? *Prompt*: your location, financial concerns.   Advice   - If you could give advice to a parent or a family who have just received a diagnosis of brain tumour for their child, what would it be? |
| Treatment – about halfway | |
| Primary care giver | *I am going to ask you some questions about how you and your family are experiencing [child’s name]’s brain tumour.*   - How has this experience impacted on your daily life? *Prompts*: paid work, social life. - How has this experience impacted on your own physical and emotional wellbeing? - How has this experience impacted on your relationship with your partner [or your child’s mother/father]? - How, do you think, [child’s name] has been impacted by this experience? *Prompts*: schooling, social life, emotional wellbeing. - How, do you think, your other children have been impacted by this experience? *Prompts*: schooling, social life, emotional wellbeing. - How, do you think, this experience has impacted on your family as a whole?   Care   - What care or services, if any, have you or your family received so far to address the aspects of your experiences we have just discussed? - What are some other things that you or someone else has done to help you and your family in this experience? - What are some things, if any, that are making this experience more difficult for you or your family? - How would you like these to change? - How well, if at all, do you think different health care providers and services have worked together to provide care to your child and your family? |
| Treatment – about to finish/recently completed | |
| Primary care giver | - Please tell me some thoughts or feelings you are experiencing about [child’s name]’s treatment coming to an end.   Transitioning into survivorship   - What is the care and support that you would like for your child as they transition out of treatment? *Prompts*: school/education, social skills, mental health. - What kind of conversations and planning, if any, have taken place to ensure [child’s name] receives this treatment? - Is there anything about this planning that you think could be improved?   Support for carers   - Going forward, do you feel adequately supported in your role as primary carer of your child? - How could health or community services better support you? |
| Mother | - Please tell me what it means to you to be a mother to a child being treated for brain tumour/cancer. - Do you think you experience aspects of your child’s brain tumour/cancer and treatment differently than your partner? Please explain. - Have you ever felt excluded from your child’s care? - Is there anything that you would like people to know about parenting a child diagnosed with a brain tumour? - What advice would you give to other mothers who have a child with a brain tumour? |
| Father | - Please tell me what it means to you to be a father to a child being treated for brain tumour/cancer. - Do you think you experience aspects of your child’s brain tumour/cancer and treatment differently than your partner? Please explain. - Have you ever felt excluded from your child’s care? - Is there anything that you would like people to know about parenting a child diagnosed with a brain tumour? - What advice would you give to other fathers who have a child with a brain tumour? |
| Survivorship | |
| Primary care giver | - Please tell me about some of the things that you are feeling now that your child is no longer receiving treatment for a brain tumour.   School   - Can you please tell me about what it was like when your child went back to school. *Prompts*: - Was there anyone who reached out to you to assist with your child’s transition prior to them returning to school? - How, do you think, your child has experienced their return to school? - What, if anything, were the positive aspects of your child’s return to school? - What, if anything, were the aspects that could have been improved upon? - Is there anything else you would like to tell me about returning to school after having been treated for a brain tumour?   Social   - How would you describe your child’s social functioning (i.e. how they get along with children their age)? - How does this affect the way they may think or feel about themselves? - What are some things that help your child to socialise with other children? - What are some things that make it more difficult for your child to socialise with other children?   Other late effects   - Have there been any other changes or health concerns for your child due to their brain tumour and treatment? - Did you know any of these changes were possible or likely to happen to your child after treatment? - How and when did you learn about this? How did you feel about this?   Survivorship care   - What care and support, if any, has your child and your family been offered to help with the effects of the brain tumour and treatment? - Since your child finish treatment for their tumour, how could health and community services better support…? - Your child - You as parent and care giver - Your family - How well, if at all, do you think different health care providers and services have worked together to provide care to your child and your family now that treatment has ended?   The future   - What do you see for your child’s future? *Prompts*: Where will they be living? Working? Relationships? - How is this different to what you had envisioned for them before their diagnosis? - Do you think of your future differently compared to before your child was diagnosed? - Looking forward, what do you see for your family? - Is this different to what you had envisioned before [child’s name] was diagnosed?   For benign tumour only:   - Do you perceive there to be any differences in the experiences of children and their families who are living with a brain tumour rather than brain cancer? If yes, what are these? - Is there anything else that you would like to tell me about caring for a child who has survived a brain tumour/cancer? |
| Mother | - Please tell me what it means to you to be a mother to a child who has had treatment for a brain tumour. - Do you think you experience your child’s survivorship differently than your partner? Please explain. - Is there anything that you would like people to know about parenting a child diagnosed with a brain tumour? - What advice would you give to other mothers who have a child diagnosed with a brain tumour? |
| Father | - Please tell me what it means to you to be a father to a child who has had treatment for a brain tumour. - Do you think you experience your child’s survivorship differently than your partner? Please explain. - Is there anything that you would like people to know about parenting a child diagnosed with a brain tumour? - What advice would you give to other fathers who have a child diagnosed with a brain tumour? |
| Conclusion of each interview |  |
| All participants | - Is there anything else that you would like to tell me about? |
